# Cutaneous tuberculosis in HIV-infected individuals: Lessons learnt from a case series

**DOI:** 10.4102/sajhivmed.v20i1.895

**Published:** 2019-03-12

**Authors:** Vhudzani Tshisevhe, Nontombi Mbelle, Remco P.H. Peters

**Affiliations:** 1 Lancet Laboratories, Rustenburg, South Africa; 2 Department of Medical Microbiology, Faculty of Health Sciences, University of Pretoria, Pretoria, South Africa; 3 Department of Medical Microbiology, Tshwane Academic Division, National Health Laboratory Service, Pretoria, South Africa; 4 Department of Medical Microbiology, School for Public Health and Primary Care, University of Maastricht, Maastricht, the Netherlands; 5 Anova Health Institute, Johannesburg, South Africa

**Keywords:** HIV-medicine, Retro-Viral Disease, Mycobacteria, Tuberculosis, Multidrug Resistance, Cutaneous Tuberculosis, Cutaneous Disease, immunocompromised

## Abstract

**Introduction:**

Extrapulmonary tuberculosis (TB) causes a significant burden of disease worldwide, especially among HIV-infected individuals and those with other immunosuppressive conditions. Cutaneous TB is an important manifestation of extrapulmonary TB but is uncommonly reported in South Africa despite the high burden of HIV and TB co-infection. There is a paucity of published data on clinical presentation and outcome of cutaneous TB in this context. Raising awareness of this condition among clinicians is imperative to improve early diagnosis and optimise treatment outcomes.

**Patient presentation:**

In this series, we present three cases of cutaneous TB, two adults and one child, referred to a tertiary hospital from two primary healthcare centres and from a general practitioner. We demonstrate that the clinical presentation is diverse, ranging from papular lesions to abscesses, and that concordant pulmonary TB may be present.

**Management:**

In particular, we show the importance of performing diagnostic procedures (e.g. aspiration) in individuals presenting with an abscess that does not respond to broad spectrum antimicrobial treatment, particularly in those with advanced immunosuppression.

**Outcome and conclusion:**

The outcome of our three patients was poor, highlighting the need for earlier diagnosis in this WHO Stage 4 condition and intensive management of clinical cases.

## Introduction

Cutaneous *Mycobacterium tuberculosis* (MTB) infection is a form of extrapulmonary tuberculosis (TB) that is uncommon: it accounts for less than 2% of all cases of TB.^1,2^ The incidence of cutaneous TB has been reported to decrease in India while increasing incidence was reported in Mexico; there are no time trend data for South Africa available.^2,3,4^ The human immunodeficiency virus (HIV) epidemic is an important driver of the occurrence of cutaneous TB; other important factors are the increased rates of TB in specific settings such as healthcare facilities, prisons and homeless shelters; intravenous drug use; diabetes mellitus; and immunosuppressive therapy.^5,6,7,8,9^

The diagnosis of cutaneous TB is often challenging. Firstly, the clinical presentation of cutaneous TB is non-specific and variable. Furthermore, microbial diagnosis is complicated for reasons including the paucibacillary nature of the skin lesions, requirements for prolonged incubation period of culture in the laboratory and the difficulties in extracting and detecting mycobacteria from biopsy material.^2,5,10^ Lastly, in our experience, the awareness of cutaneous TB among health practitioners is low, which may result in delays. As a result, cutaneous TB is often diagnosed relatively late and may have poor outcomes, despite the availability of effective anti-TB treatment.

There is a paucity of data on the clinical and microbiological features of cutaneous TB and its clinical outcomes in sub-Saharan Africa. To improve treatment outcomes, increased awareness of this condition and the appropriate diagnostic work-up among healthcare workers is required. Here, we describe three cases of cutaneous TB and present lessons learnt to improve daily clinical practice.

## Cases

### Case 1

A 34-year-old HIV-infected man, who had been on antiretroviral therapy (ART) for eight months, presented to hospital with a skin abscess on his lower back of more than 1 month’s duration. He had been treated with amoxicillin-clavulanic acid at a nearby primary healthcare (PHC) facility on first presentation. Upon return to the PHC facility one month later, the abscess had increased in size, and the patient was referred to the hospital. Aspirates were taken from the abscess and submitted to the laboratory of medical microbiology, where both the Xpert® Mycobacterium tuberculosis (MTB)/Rif and line probe assay detected *M. tuberculosis*. Based on these two tests, the strain was determined to be resistant to rifampicin and sensitive to isoniazid. Second-line drug susceptibility testing revealed that the strain was sensitive to second-line injectable drugs and fluoroquinolones. Of note is that the patient completed treatment for drug-sensitive pulmonary TB 6 months prior to presenting with the skin abscess and had no concomitant signs or symptoms of pulmonary TB when he presented with the skin abscess. It is not known if the pulmonary TB was cured. His CD4+ count was 122 cells/µL when ART was initiated and 30 cells/µL at the time of cutaneous TB diagnosis. He was initiated on kanamycin, moxifloxacin, ethionamide, terizidone, ethambutol, isoniazid and pyrazinamide for cutaneous rifampicin-resistant TB (regimen used in 2016). The patient died at home two weeks after TB treatment initiation; cause of death could not be determined.

### Case 2

A 21-month-old male paediatric patient on ART for eight months presented with multiple abscesses on the forearms and torso. His mother was on drug-sensitive TB treatment at the time of the patient’s presentation, and a year prior, the father had died of pulmonary TB. The patient received bacillus Calmette-Guérin vaccine at birth. He had first presented at the general practitioner (GP) with a week’s history of intermittent cough, papular lesions on the torso and failure to thrive. The treatment given by the GP included amoxicillin, trimethoprim-sulfamethoxazole and vitamin B complex syrup. Gastric aspirates tested negative for TB with Ziehl-Neelsen and mycobacteria growth indicator tube (MGIT) culture. The patient returned to the GP a month later, and the papular lesions had formed abscesses and extended to the forearms. The patient was transferred to the hospital for further management. Mycobacteria growth indicator tube culture of the abscess aspirate grew *M. tuberculosis*, and line probe assay confirmed the organism as sensitive to rifampicin and isoniazid. Two respiratory specimens collected at different times after cutaneous TB diagnosis tested negative for *M. tuberculosis*. Hospital records showed poor adherence to HIV treatment as evident by HIV viral load of 260 000 copies/mL six months after ART initiation and missed follow-up appointments. The patient was initiated on rifampicin, isoniazid, pyrazinamide and ethambutol for cutaneous TB. He died four weeks after starting treatment from respiratory tract infection, the cause of which was not determined, as the patient died on arrival at the hospital and no autopsy was done.

### Case 3

A 42-year-old HIV-positive female patient who had never been on ART presented to her local hospital with a 2-month history of a non-healing cold abscess on the right forearm. The patient had been treated with flucloxacillin and trimethoprim-sulfamethoxazole by her local GP, whom she consulted on three occasions prior to hospital presentation. Incision and drainage was also done by the GP during her last visit. Abscess aspirates were collected and submitted to a microbiology laboratory, where *M. tuberculosis* was detected by MGIT culture and confirmed by line probe assay. The patient was initiated on a first-line anti-TB regimen: rifampicin, isoniazid, pyrazinamide and ethambutol. Pulmonary TB was also diagnosed a month later following new onset of cough, loss of weight and a positive Xpert® test on sputum. Both the cutaneous and pulmonary *M. tuberculosis* strains were susceptible to rifampicin and isoniazid; and the initial treatment was continued following the diagnosis of pulmonary TB. The patient was lost to follow-up and thus the outcome is unknown.

## Ethical consideration

Ethical approval was obtained from the Faculty of Health Sciences Research Ethics Committee of the University of Pretoria (ethics reference number: 145/2018).

## Discussion

Cutaneous TB can be acquired through direct infection of the skin (exogenous TB) or from haematogenous spread of TB elsewhere in the body.^11^ It may not always be obvious how the patient acquired the cutaneous TB. Haematogenous spread of TB may be the most likely cause in the first patient, as he had recently been treated for pulmonary TB and presented with low CD4+ count, putting him at increased risk for disseminated infection. Direct infection of the skin could have occurred in the second patient, although haematogenous spread of asymptomatic pulmonary infection is more likely because children are more prone to disseminated TB. The mode of acquisition is unclear in the third patient. It is clinically important, however, to assess patients with cutaneous TB for the presence of pulmonary infection, especially because these infections may be caused by strains with different resistance profiles.^12^

Despite the strong association with HIV, cutaneous TB is rarely reported in South Africa, the country with the largest HIV–TB epidemic in the world.^11,13^ Most likely, under-diagnosis and under-reporting occur because of the limited awareness of this condition. The clinical presentation of cutaneous TB is non-specific and variable. The cases presented illustrate that skin lesions may occur on different parts of the body, can be single or multiple and could evolve from papular lesions to abscesses.^14^ All patients had initially been treated with broad spectrum antibiotics, without success. Correctly, the clinicians referred these patients to the hospital for further diagnostics and management, where cutaneous TB was confirmed through abscess aspiration. It is noteworthy that tissue biopsies can also be used for cutaneous TB diagnosis using Xpert® MTB/Rif.^15^ These cases highlight the importance of sampling any lesion that does not respond to initial broad spectrum antimicrobial treatment. As we show in our case series, this holds particularly true for those at highest risk of cutaneous TB: HIV-infected individuals with advanced immunosuppression.^6^ The same applies to cutaneous lesions in children, as infancy is another known risk factor that may be further enhanced by other individuals with pulmonary TB in the household.^5,14,16,17^ As such, a diagnosis of cutaneous TB is an important indicator for further investigations and management of other conditions, in particular HIV-associated advanced immunosuppression.

While our cases presented with abscesses; it is important to note that cutaneous TB can present in various forms depending on bacillary load. The varieties include the multibacillary forms such as tuberculous chancre, scrofuloderma, orificial TB and tuberculous gumma and paucibacillary forms such as verrucosa cutis and lupus vulgaris.^5,16^ There are also skin lesions associated with immune hypersensitivity reactions to *M. tuberculosis* antigens. These lesions are of three variants, which include papulonecrotic tuberculid, lichen scrofulosorum and erythema induratum.

The outcome of cutaneous TB is rarely reported in the literature, but two of our patients died and the third was lost to follow-up. However, cutaneous TB generally occurs in a vulnerable group because of high level of immunosuppression, young age and various comorbidities. Intensive follow-up of TB care as well as intensive management of all other underlying conditions is required to ensure optimal outcome.

## Conclusion

The diverse clinical presentation of cutaneous TB requires a high index of suspicion when patients present with skin lesions that do not respond to broad spectrum antibiotics, especially in the presence of other risk factors such as HIV infection. Abscess aspiration is an essential diagnostic procedure.
